# Resolutions on the Death of Dr. Theodore Menges

**Published:** 1900-10-15

**Authors:** Lloyd R. Hawley, W. C. Skidmore, Louis Ottofy

**Affiliations:** Committee. Manila, P. I.; Committee. Manila, P. I.; Committee. Manila, P. I.


MANILA, P. I., August 6, 1900.

### RESOLUTIONS ON THE DEATH OF DR. THEODORE MENGES.

The Manila Dental Society, numbering among its members former associates and pupils of Dr. Theo. Menges, have learned with great sorrow of his demise while yet in the prime of manhood, and at a meeting held this day have adopted the following resolution, of which a copy is ordered to be spread upon the minutes of the Society, one to be sent to his sorrowing widow and others to the dental journals of the United States:

WHEREAS, Our departed friend, by his untiring energy, by his constant devotion to duty, has done more than his share for the uplifting of dental education in the United States; and,

WHEREAS, A host of his former pupils have each and all lost not merely a teacher but a true friend, adviser and counselor, whose constant aim was the betterment of their condition; now, therefore, be it

*Resolved*, That in his death the dental profession has lost one whose efforts on her behalf have always been elevating, his former pupils have lost a worthy friend, his wife has been bereaved of a loving husband and constant companion; that with all of these we sympathize and mourn, keenly feeling while far away from our homes and friends, that in his passing away, we too, have lost.

LLOYD R. HAWLEY,

W. C. SKIDMORE,

LOUIS OTTOFY, } *Committee.*

